# Efficacy and Mechanism of Transcutaneous Auricular Vagus Nerve Stimulation in Patients With Poststroke Dysphagia: Protocol for a Randomized, Sham-Controlled Clinical Trial

**DOI:** 10.2196/95182

**Published:** 2026-10-28

**Authors:** Luming Gao, Jin Li, Miao Xie, Canyi Xu, Changying Zhang, Weichuan Kuang, Xi Wen, Xiaoyin Wang, Yue Liu, Yu Wang, Tong Liu

**Affiliations:** 1 The Fifth College of Clinical Medicine, Guangzhou University of Traditional Chinese Medicine, Guangzhou, China; 2 Guangdong Provincial Engineering Technology Research Institute of Traditional Chinese Medicine, Guangzhou, Guangdong, China; 3 Guangdong Provincial Key Laboratory of Research and Development in Traditional Chinese Medicine, Guangzhou, Guangdong, China; 4 Department of Acupuncture and Rehabilitation, Guangdong Provincial Second Hospital of Traditional Chinese Medicine, 60 Hengfu Road, Yuexiu District, Guangzhou, Guangdong, China, 86 18319673139; 5 Institute of Acupuncture and Moxibustion, China Academy of Chinese Medical Sciences, Beijing, Beijing, China

**Keywords:** transcutaneous auricular vagus nerve stimulation, poststroke dysphagia, stroke, protocol, randomized controlled trial

## Abstract

**Background:**

Dysphagia is a common complication following stroke. While current management strategies for poststroke dysphagia (PSD) range from nutritional support to neurostimulation techniques, an optimal treatment regimen remains elusive. As a promising noninvasive neuromodulation approach, transcutaneous auricular vagus nerve stimulation (taVNS) requires further validation in rigorously designed trials to establish robust evidence of its efficacy and mechanisms of action.

**Objective:**

This study aims to validate the efficacy of taVNS in treating PSD and to elucidate its underlying mechanisms, thereby supporting the development of a novel, noninvasive therapeutic strategy for improving swallowing function in stroke survivors.

**Methods:**

This study will use a prospective, randomized, double-blind, controlled trial design. A total of 40 patients with PSD will be enrolled and randomly allocated to either a taVNS group or a sham taVNS group. Both groups will receive conventional swallowing rehabilitation therapy as the baseline intervention. The taVNS group will receive bilateral electrical stimulation at the cymba conchae and cavum conchae of the auricle. These sites correspond to the “Heart” and “Kidney” auricular acupoints, selected in accordance with traditional Chinese medicine theory. In contrast, the sham taVNS group will receive stimulation at regions of the auricle not innervated by the vagus nerve. The primary outcome measure will be the Penetration-Aspiration Scale score. Secondary outcomes will include the Yale Pharyngeal Residue Severity Rating Scale, hyoid bone displacement and hyoid-thyroid approximation distance assessed via B-mode ultrasonography, and motor-evoked potentials of the suprahyoid muscles elicited by transcranial magnetic stimulation. All outcome measures will be evaluated at baseline and after the 2-week intervention period.

**Results:**

This paper describes the protocol for a study evaluating the efficacy and mechanisms of taVNS in patients with PSD. The study was funded in January 2025. Participant recruitment began in March 2025 and is projected to conclude by October 2026. Data collection began in March 2025 and is projected to be completed by October 2026. Primary study results are expected to be submitted for publication in winter 2026.

**Conclusions:**

This study aims to validate the efficacy of taVNS in treating PSD and to elucidate its underlying mechanisms, thereby supporting the development of a novel, noninvasive therapeutic strategy for improving swallowing function in stroke survivors.

## Introduction

Stroke is a prevalent cerebrovascular disease with high incidence, disability, and mortality. Dysphagia, one of its most frequent complications, occurs in 37% to 78% of patients [1] and can severely compromise nutritional status, quality of life, and overall prognosis.

Current management of poststroke dysphagia (PSD) primarily involves nutritional and oral hygiene support, pharmacotherapy, behavioral therapy, and neurostimulation techniques [2]. Although noninvasive peripheral and central nervous system stimulation methods—such as pharyngeal electrical stimulation, transcranial magnetic stimulation (TMS), and transcranial direct current stimulation—have gained research attention and shown promising results [3], yet significant heterogeneity across studies remains, and no optimal treatment protocol has been established. Thus, there is a need to develop additional therapeutic approaches to enhance rehabilitation outcomes and achieve satisfactory functional recovery in PSD.

The vagus nerve plays a critical role in swallowing. Its fibers innervate both striated and smooth muscles, not only controlling motor function of swallowing-related muscles but also regulating activities in other swallowing-associated organs, such as respiratory movements in the lungs [4]. Vagus nerve stimulation can directly enhance the swallowing reflex and modulate the involved musculature. Existing preliminary evidence suggests that transcutaneous auricular vagus nerve stimulation (taVNS) may facilitate swallowing recovery via activation of the auricular vagal branch, which could engage central vagal-swallowing neural circuits and potentially drive corticobulbar tract functional reorganization; however, robust mechanistic evidence from rigorously controlled double-blind trials remains insufficient. For instance, Wang et al [5] conducted a randomized sham-controlled trial in 40 patients with acute PSD, demonstrating that taVNS promotes swallowing recovery in acute stroke. However, their study was limited to patients with acute stroke, and the sham stimulation—applied without electrical current—was susceptible to unblinding, as participants could detect the absence of somatic sensation, thus compromising methodological rigor. To address these limitations, we developed a more robust protocol by enrolling patients in the stroke recovery phase and incorporating an active control group. This control group receives stimulation with parameters identical to those of the taVNS group but applied to regions outside vagal innervation zones, thereby improving the validity and completeness of our experimental design.

The theoretical framework of this study integrates principles from traditional Chinese medicine (TCM) with modern Western medical science. The research design is based on the theory of holographic embryology, which suggests that the auricle represents a microsystem that reflects all organs and parts of the human body [6]. This concept is supported by the classical TCM text Spiritual Pivot (Questioning), which states that “the ear is the site where the ancestral vessels converge,” emphasizing its role as a convergence point of meridian qi and a window for pathological manifestations. According to TCM theory, sufficient Heart Qi ensures flexible tongue movement conducive to swallowing, while adequate Kidney Qi supports mental clarity, thereby promoting swallowing function. Therefore, the experimental protocol focuses on stimulating the Heart zone (located in the cymba conchae) and the Kidney zone (situated in the cavum conchae) of the auricle.

Modern neuroanatomy has established that the concha is the sole region on the body surface containing afferent vagus nerve fibers [7]. Notably, the anatomical locations of the Heart and Kidney auricular points converge within these vagal projection zones. This anatomical overlap suggests that electroacupuncture at these points may activate neural pathways analogous to those engaged by direct vagus nerve stimulation [8]. The resulting neural impulses are projected via afferent fibers to the central nervous system, where they may modulate neuronal activity through changes in electroencephalographic rhythms and neurotransmitter levels. This mechanism is implicated in the treatment of brain disorders [9] and, from a Western medical standpoint, may also facilitate improvement in swallowing function.

## Methods

### Trial Design

This study will use a prospective, randomized, double-blind, controlled trial design to evaluate the efficacy and mechanisms of taVNS in promoting recovery in patients with PSD. After written informed consent is obtained from participants and their guardians, baseline demographic and clinical characteristics—including vital signs, neurological history, surgical and medication records, and electrocardiograms—will be collected in accordance with the trial’s inclusion and exclusion criteria.

Swallowing function will be evaluated using a combination of instrumental and clinical scales: the Penetration-Aspiration Scale (PAS), Fiberoptic Endoscopic Evaluation of Swallowing (FEES), Yale Pharyngeal Residual Severity Rating Scale (YPR-SRS), Modified Mann Assessment of Swallowing Ability (MASA), and the Chinese version of the Functional Oral Intake Scale (FOIS). Hyoid bone displacement and hyoid-thyroid approximation distance during volitional swallowing will be quantified using B-mode ultrasonography. Furthermore, motor-evoked potentials (MEPs) of the suprahyoid muscles will be recorded using a transcranial magnetic stimulator equipped with an MEP detection module (TMS-MEP).

Participants will be randomly assigned to either a taVNS group or a sham taVNS group, with 20 participants in each group. All participants will receive conventional swallowing rehabilitation in addition to their assigned stimulation intervention. Vital signs will be monitored after each session, and any adverse events (AEs) will be recorded. The FOIS will be reassessed at the 1-month follow-up. The trial flowchart is shown in Figure 1, and this trial protocol was developed in accordance with the SPIRIT (Standard Protocol Items: Recommendations for Interventional Trials) reporting guideline (Checklist 1).

**Figure 1.**
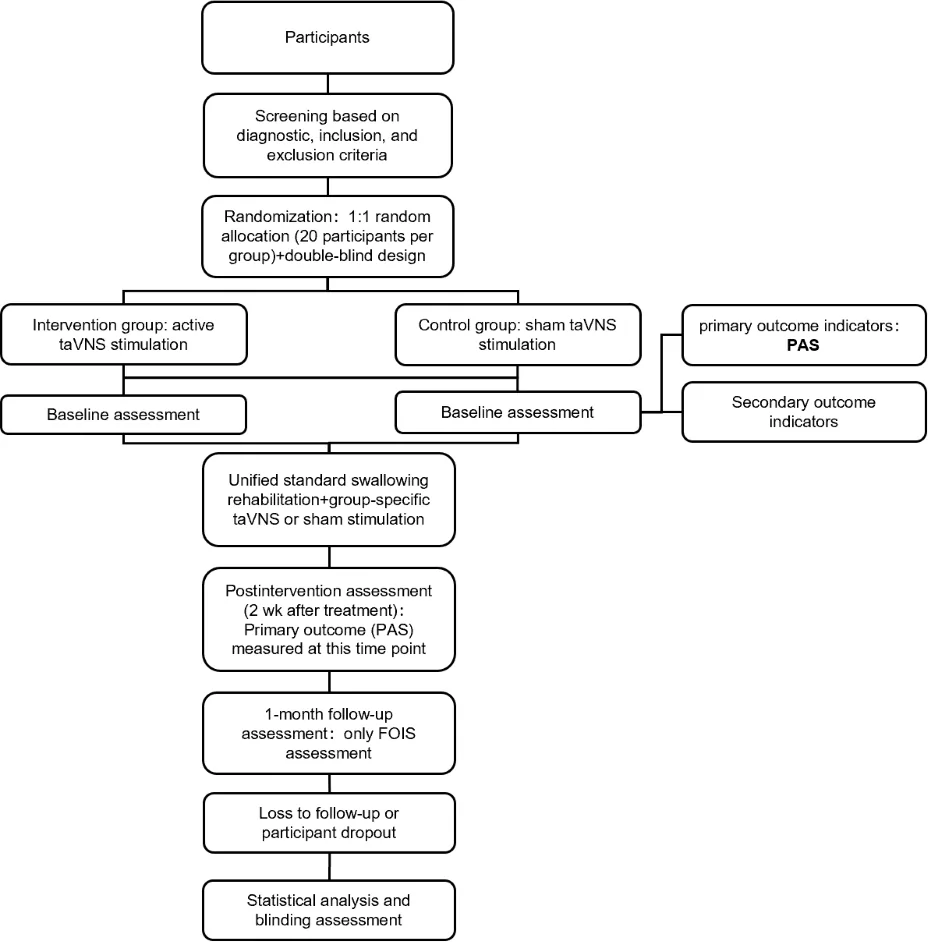
Study flow diagram. After screening and enrollment, 40 participants will be randomized 1:1 to receive either taVNS plus standard rehabilitation or sham taVNS plus standard rehabilitation. Outcomes will be assessed at baseline (day 0), postintervention (day 14), and follow-up (day 44). FOIS: Functional Oral Intake Scale; PAS: Penetration-Aspiration Scale; taVNS: transcutaneous auricular vagus nerve stimulation.

### Objectives

This study aims to evaluate the clinical efficacy of taVNS in treating PSD and to investigate the underlying mechanisms by which taVNS improves swallowing function.

### Participants

#### Study Setting

Eligible participants will be recruited from the Acupuncture and Rehabilitation Department of Guangdong Second Hospital of Traditional Chinese Medicine. All participants must have a clinical and imaging-confirmed diagnosis of PSD and provide written informed consent prior to enrollment. The detailed inclusion and exclusion criteria are presented in Textbox 1.

Textbox 1. **Inclusion and exclusion criteria.**

**Inclusion criteria**

- Participants aged 18-70 years, regardless of gender
- A score of 11.3 on the Kubota Water Swallowing Test or impaired swallowing safety as assessed by the Volume‑Viscosity Swallow Test—Chinese Version
- Duration of dysphagia between 2 wk and 6 mo
- Relatively stable medication regimen for at least 1 week prior to enrollment
- Signed informed consent

**Exclusion criteria**

- Patients with unstable vital signs or with active cerebral hemorrhage
- Documented cardiac arrhythmia or baseline heart rate <60 beats/min
- Presence of implantable medical devices, such as cardiac pacemakers, intracranial metallic implants, or cochlear implants
- Local contraindications to electrical stimulation, including active external ear infection, dermatological lesions, or structural malformations
- History of vagus nerve surgery or traumatic injury
- Dysphagia attributable to nonstroke etiologies
- Significant cognitive impairment or consciousness disorders that may compromise assessment compliance

#### Elimination Criteria

Elimination criteria included poor compliance and unwillingness to cooperate with outcome assessment, or poor compliance and a refusal to cooperate with the implementation of interventions.

#### Dropout Criteria

Dropout criteria included poor adherence to the study protocol, specifically refusal to cooperate with outcome assessments, or unwillingness to comply with the assigned intervention regimen.

#### Discontinuation Criteria

Discontinuation criteria included the occurrence of severe complications or an active systemic infection during the trial period, or the emergence of severe allergic reactions or other serious AEs attributable to the intervention.

#### Randomization

Prior to study initiation, all patients will receive a comprehensive explanation of the trial procedures, including expected time commitment, potential benefits, and foreseeable risks. Written informed consent will be obtained from each participant prior to enrollment. A total of 40 randomization cards indicating group assignment will be prepared in advance according to a computer-generated randomization sequence. Each card will be sealed inside an opaque envelope of uniform size and color. These envelopes will be securely stored and managed by an independent member of the research team who is not involved in patient recruitment or outcome assessment. As eligible participants are enrolled, a designated study specialist will open the envelopes sequentially in the order of enrollment and implement the intervention allocation specified on the card. This procedure will result in the random allocation of 40 participants to 1 of 2 groups. All participants will receive standard medical management for PSD. Those assigned to the intervention group will additionally receive active taVNS, while participants in the control group will receive sham taVNS alongside standard care.

#### Blinding

This study adopts a double-blind design: participants, all outcome assessors (including the neuro-otolaryngologist for FEES, ultrasonographer, and TMS operator), and statistical analysts remain blinded to group assignment. Therapists delivering taVNS or sham stimulation are the only staff aware of the allocation and are excluded from all outcome measurement and data analysis procedures.

Identical stimulators matching in appearance, weight, indicator lights, and sound prompts are used for both groups. Both the taVNS and sham taVNS groups receive real electrical stimulation with a fully unified set of parameters (waveform, frequency, adjustable intensity range, single-session duration, and weekly treatment frequency). The only difference between groups is the stimulation site: the taVNS group receives stimulation at the vagal-innervated concha region (Heart CO15 and Kidney CO10 acupoints), while the sham group receives stimulation at nonvagal scapha acupoints (Elbow SF3, Shoulder SF4-5), which lie outside the vagus nerve innervation zone. This design ensures that both groups experience identical cutaneous sensations, thereby maintaining effective blinding while avoiding vagal activation in the sham group. To mitigate unblinding risks stemming from inherent sensory differences between concha and scapha stimulation sites, targeted masking strategies are implemented. Stimulation intensity is adjusted per participant to achieve an equivalent mild tingling sensory experience at the respective auricular locations. All stimulation sessions are conducted in separate single-person treatment rooms, and participants cannot see electrode placement. Therapists follow standardized, neutral verbal guidance and avoid differential descriptions of stimulation sensations to prevent suggestive bias.

Blinding efficacy will be quantitatively assessed after the trial’s completion using the Bang Blinding Index (BBI), which is based on participants’ postintervention group guesses, as detailed in the Statistical Analysis section. Participants’ self-reported stimulation sensory experience will be collected for post hoc analysis to detect site-related sensory bias. Considering the anatomical differences between vagal and nonvagal auricular regions, between-group efficacy results will not be interpreted as purely vagus nerve–specific effects without confirmation of adequate blinding via the BBI.

Emergency unblinding is reserved exclusively for the independent safety officer when serious AEs occur that require targeted clinical intervention. All unblinding incidents, root causes, and timelines will be fully documented in the medical records and submitted to the institutional ethics committee in a timely manner.

#### Sample Size Calculation

The study design was adapted from the methodology used by Wang et al [5]. Based on baseline data from that study, the PAS scores for the 2 groups were reported as 2.89±0.46 and 3.60±0.68, respectively. Sample size estimation was conducted using G*Power software (version 3.1.7) with a 2-tailed analysis of variance model. The analysis assumed a significance level (α) of .05, statistical power of .90 (β=.10), and the intergroup difference in PAS scores observed in the prior study. The calculation indicated that 16 participants per group were required, yielding an initial total sample size of 32. To accommodate a potential 15% dropout rate, the sample size was adjusted to 38 participants. In alignment with the sample size used in the study by Wang et al [5], a total of 40 patients were ultimately enrolled.

### Ethical Considerations

This study will be conducted in accordance with the Declaration of Helsinki and Good Clinical Practice guidelines. Ethical approval for this study was obtained from the Research Ethics Committee of the Guangdong Second Hospital of Traditional Chinese Medicine on January 2, 2025 (Approval Number: Y202411-003-01). The trial was registered with the Chinese Clinical Trial Registry on January 21, 2025 (ChiCTR2500096311).

Written informed consent will be obtained from all participants prior to enrollment. Before recruitment, researchers will explain the study’s purpose, procedures, potential risks, and benefits to potential participants and their guardians. Participants will be informed that they may withdraw from the study at any time without affecting their medical care.

Data confidentiality will be maintained throughout the study. All participant-identifying information will be anonymized before analysis [10]. Study data will be securely archived and managed by the principal investigator for a period of 3 years following study completion, in accordance with institutional policies. Deidentified datasets generated in this study may be made available from the corresponding author upon reasonable request [11,12].

Any amendments to the study protocol will require unanimous agreement from the research team and will be formally documented by updating the clinical trial registry [13].

The Clinical Endpoints Committee consists of members of the Data Monitoring Committee and principal investigators who will review source data and medical events based on prespecified outcomes. The Steering Committee consists of the principal investigator, subinvestigators, rehabilitation department directors, and methodologists. The committee holds routine quarterly meetings to review recruitment progress, the incidence of adverse events, problems with protocol compliance, and any proposed protocol amendments. Any plans for trial adjustments must obtain unanimous written approval from all committee members before submission to the clinical trial registry and the ethics committee.

### Interventions

#### Standard Therapy

##### Pharmacological Therapy

All enrolled participants will receive standard pharmacological treatment for stroke in accordance with the Chinese Stroke Prevention and Treatment Guidelines (2021 Edition) [10,14]. The medication regimen will include, but not be limited to, the following: active agents for secondary prevention, cerebral circulation enhancers, neuroprotective drugs, cognitive enhancers, antidepressants, anxiolytics, antiepileptics, and muscle relaxants.

##### Physical Rehabilitation Therapy

Patients will undergo daily sessions of physical and occupational therapy, each lasting 30 minutes.

The physical therapy protocol will comprise passive range-of-motion exercises, turning and repositioning training, muscle-strengthening activities, balance retraining, ambulation practice, and a range of low- to medium-frequency electrotherapies.

Occupational therapy will aim to enhance functional independence through activities of daily living training, joint mobility exercises, roller-based coordination activities, and ball-throwing tasks.

##### Swallowing Rehabilitation Therapy

Behavioral swallowing therapy includes lip and tongue strength training, compensatory techniques for airway protection during swallowing, and cold thermal stimulation. Neuromuscular electrical stimulation is delivered via the VitalStim 590 swallowing therapy device (Enovis), targeting the suprahyoid and thyrohyoid muscles for 20 minutes per session, once daily.

All standard therapy components will be provided identically to all participants in both the taVNS and sham taVNS groups. The dose of each therapy component is standardized:

- Pharmacological therapy was administered as per guidelines; dose and frequency were recorded for each participant.
- Physical rehabilitation consisted of 30 minutes daily, 5 days a week.
- Occupational therapy consisted of 30 minutes daily, 5 days a week.
- Swallowing rehabilitation consisted of 20 minutes daily, 5 days a week.

Adherence to standard therapy will be recorded in daily therapy logs. Participants will be considered adherent if they complete at least 80% of scheduled therapy sessions. Therapy logs will be reviewed weekly by the study coordinator.

### taVNS and Sham Stimulation

Patients assume a seated position. Before stimulation, the auricular sites and adjacent skin are disinfected with alcohol swabs. A Huatuo SDZ-IIB electronic stimulator (Suzhou Medical Appliance Factory Ltd), equipped with dedicated leads for channels A and B, is used to apply 2 pairs of auricular clips bilaterally to the cymba conchae and cavum conchae regions. Stimulation parameters are configured as follows: a dense-sparse wave mode (alternating between 4 Hz for 5 s and 20 Hz for 10 s), with an intensity ranging from 0.6 to 1.0 mA, adjusted in real-time according to patient tolerance. In the taVNS group, stimulation targets the Heart (CO15, located in the cavum conchae) and Kidney (CO10, situated in the cymba conchae) acupoints [15], which were selected based on their anatomical positions within the vagus nerve innervation area. Both groups receive active electrical stimulation; the groups differ only in stimulation location. In contrast, the sham taVNS group receives stimulation at the Elbow (SF3) and Shoulder (SF4-5) acupoints on the scapha [16], which lie outside the vagus nerve innervation zone, thereby controlling for nonspecific effects. The treatment is administered twice daily (morning and afternoon), with each session lasting 30 minutes. Sessions are delivered 5 days a week for a total intervention period of 2 weeks (Figure 2).

**Figure 2.**
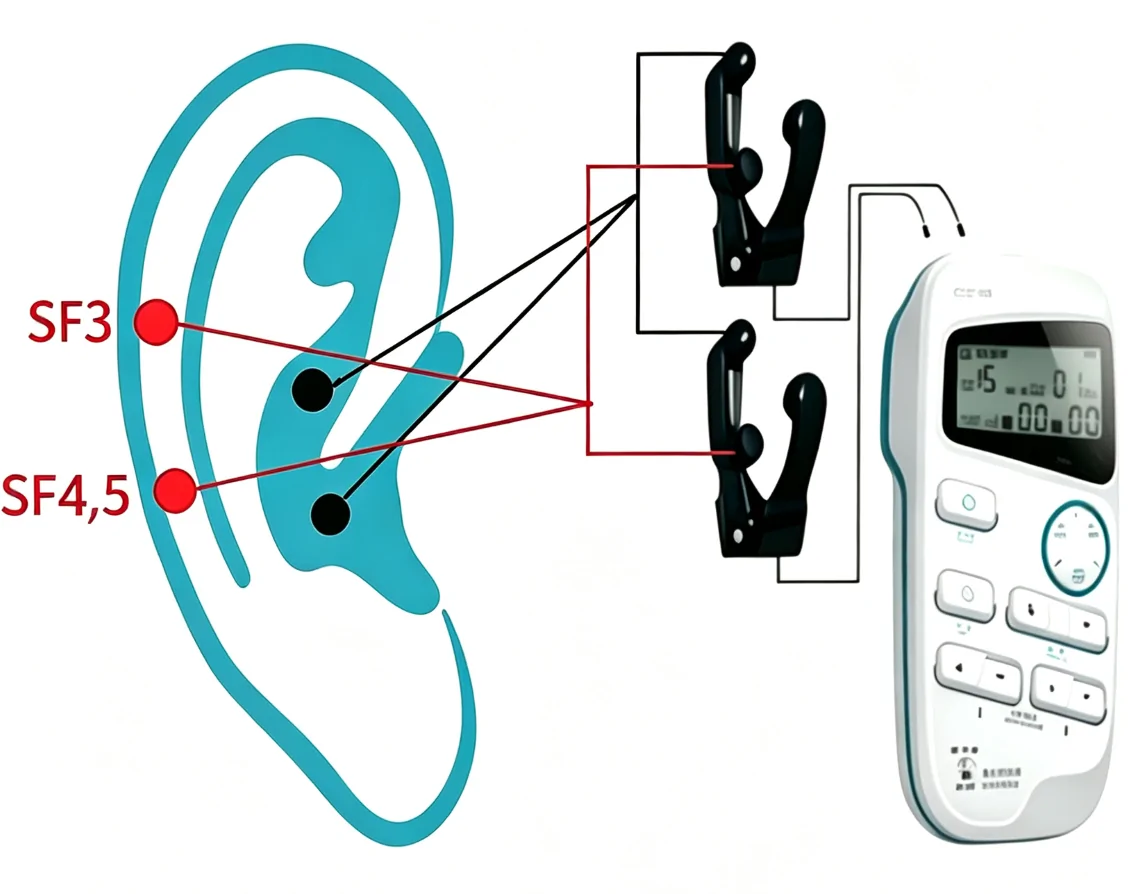
Annotated schematic of auricular anatomical regions. Solid black circles indicate active stimulation sites at the cymba conchae (CO10) and cavum conchae (CO15), where auricular vagal nerve branches are densely distributed. Solid red circles mark sham stimulation sites at elbow (SF3) and shoulder (SF4-5) points within the scapha, a vagal nerve–free zone to achieve sensory matching for the double-blind design. Standard auricular anatomical landmarks are labeled throughout the diagram.

### Provisions for Posttrial Care

Upon completion of the full intervention protocol, participants will receive a detailed report comparing their baseline clinical measures with posttreatment outcomes, accompanied by evidence-based recommendations aimed at reducing the risk of adverse clinical events.

### Outcomes

#### Primary Outcome

The primary outcome will be assessed using the Rosenbek PAS [17], which is the most widely adopted standardized instrument in clinical research for quantifying the depth of airway invasion (penetration and aspiration). The PAS is scored based on findings from the FEES procedure.

Penetration is defined as the entry of liquid or food into the laryngeal vestibule, but not beyond the vocal folds. Aspiration, in contrast, refers to the passage of material below the vocal folds into the trachea and lungs. It is further classified as overt aspiration—accompanied by coughing or choking—or silent aspiration, which occurs in the absence of such protective reflexes.

The PAS classifies aspiration severity into 8 levels according to the depth of material invasion, its position relative to the vocal folds, and the patient’s ability to clear the airway. A score of 1 indicates that no material enters the airway, while a score of 8 represents material passing below the vocal folds with no effort to eject it [17,18].

#### Secondary Outcomes

##### YPR-SRS

Pharyngeal residue is defined as the incomplete clearance of both preswallow secretions and postswallow food remnants retained in the vallecula and pyriform sinuses [18]. YPR-SRS is a standardized, image-based, 5-point ordinal scale that assesses residue severity based on anatomical location. Key metrics include the site of residue—whether in the valleculae, pyriform sinuses, or both—and the amount of residue, rated as none, trace, mild, moderate, or severe [19]. When used alongside the PAS, the YPR-SRS enables a more comprehensive evaluation of swallowing function by capturing both pharyngeal residue and aspiration events.

##### MASA

MASA is a comprehensive tool widely used to assess swallowing function. It comprises multiple items evaluating key components of the swallowing process, such as mastication, swallowing coordination, and esophageal transit. Each item is scored based on standardized criteria, with a maximum total score of 100 points. The overall score is calculated as the sum of all individual items, where higher scores indicate swallowing function that is closer to normal [17,18].

##### Hyoid Bone Displacement Distance and Hyoid-Thyroid Cartilage Approximation Distance During Volitional Swallowing

###### Hyoid Bone Displacement

A color Doppler ultrasound system (Wisonic Clover) with a 3 to 10 MHz curved array transducer will be used for hyoid movement assessment. The probe will be positioned sagittally along the midline below the mandible, covering the hyoid bone to visualize the tongue and hyoid dynamics during swallowing. Using the mandible as the coordinate origin, hyoid displacement will be quantified by measuring the coordinate difference between the resting position and the position at the peak of swallowing [20]. An increase in hyoid displacement indicates enhanced contractile strength of the suprahyoid muscles, which are responsible for laryngeal elevation [21].

###### Hyoid-Thyroid Approximation

The identical probe will be positioned longitudinally along the midline, anterior to the larynx, with its superior aspect aligned over the hyoid bone and its inferior aspect covering the thyroid cartilage. Ultrasonic imaging will be performed during swallowing to quantify the extent of hyoid-thyroid approximation. An increase in the approximation distance reflects enhanced activation of the muscles involved in elevating the laryngeal complex [22].

### TMS-MEPs of Suprahyoid Muscles: Resting Motor Threshold, Latency, and Amplitude

A transcranial magnetic stimulator (M-100; Shenzhen Yingzhi Technology) equipped with a figure-of-eight coil (BY90A; Shenzhen Yingchi Technology) and an integrated MEP module was used for synchronous signal recording [23]. Participants were instructed to maintain full muscular relaxation throughout the procedure. Surface recording, reference, and ground electrodes were positioned over the anterior belly of the contralateral digastric muscle, the angle of the mandible, and the spinous process of the sixth cervical vertebra (C6), respectively.

The TMS coil was positioned by the operator at a 45° angle relative to the cranial sagittal midline over the suprahyoid cortical hotspot. The left suprahyoid hotspot was localized 3.0±0.8 cm anterior and 4.1±0.9 cm lateral to the Cz point, while the right hotspot was identified 2.8±0.7 cm anterior and 4.0±0.7 cm lateral to the Cz point. Resting motor threshold (RMT) was determined through single-pulse stimulation. The stimulation intensity was then set to 120% of RMT, and multiple stimuli were delivered at randomized intervals. Five clearly reproducible MEP waveforms with the largest amplitudes were selected, and their latency and amplitude values were averaged for analysis.

MEP latency was defined as the time interval (in milliseconds) from cortical stimulation to the onset of the compound muscle action potential in the contralateral suprahyoid muscles.

MEP amplitude was defined as the peak-to-peak voltage (in millivolts), measured as the potential difference between the most negative and most positive peaks.

Reduced latency and increased amplitude are indicative of improved neural conduction velocity and enhanced cortical excitability. In this trial, TMS-MEP recordings were obtained bilaterally from the suprahyoid cortical areas to enable subgroup analyses and to examine the effects of taVNS on corticobulbar tract excitability.

### FOIS

The FOIS uses a 7-point scale that evaluates a patient’s functional oral intake by assessing their feeding methods and food consistency. Higher scores correspond to better oral intake ability [24].

### Outcome Assessment Procedures

All outcome assessments are carried out under uniform, standardized environmental and testing conditions to ensure measurement consistency. All trained assessors are fully blinded to group allocation and strictly independent of the therapists delivering taVNS or sham stimulation; no information about participant group assignment is shared between assessment and intervention staff.

#### FEES and PAS/YPR-SRS Scoring

FEES will be performed by a trained neuro-otolaryngologist who is blinded to group allocation. All FEES procedures will be video-recorded in their entirety.

The PAS and YPR-SRS will be scored independently by 2 trained assessors (both blinded to group allocation and to each other’s ratings) using the stored video recordings. Each assessor will score all recordings in a randomized order. If the 2 assessors’ scores disagree by more than 1 point on the PAS (or by >1 grade on the YPR-SRS), a third blinded assessor will review the recording and make the final decision. For disagreements of 1 point or less, the 2 assessors will discuss and reach a consensus.

#### Ultrasound and TMS-MEP Assessments

Ultrasound assessments (hyoid bone displacement and hyoid-thyroid approximation) will be performed and measured by a trained ultrasonographer blinded to group allocation. TMS-MEP recordings will be performed and analyzed by a trained technician blinded to group allocation.

#### Primary Time Point

The primary time point for hypothesis testing is day 14 (immediately after the 2-week intervention period). The primary comparison is the difference in PAS scores between the taVNS and sham taVNS groups on day 14, adjusted for baseline PAS score.

#### Exploratory Outcomes

Mechanistic outcomes (TMS-MEP parameters and hyoid movement measures) are considered exploratory. These outcomes will be analyzed descriptively and used to generate hypotheses for future research rather than to confirm mechanistic pathways.

### Data Collection

In addition to the study intervention, patients will complete a series of assessments and data collection activities at specified time points: during enrollment (days −3 to −1), at allocation (days −1 to 0), and on days 14 and 44. The schedule for these procedures is detailed in Table 1.

**Table 1.** SPIRIT^a^ schedule of enrollment, interventions, and assessment^b^.

	Study period			
	Enrollment	Allocation	Postallocation	
Time point (d)	–3 to –1	–1 to 0	14	44
Enrollment				
Eligibility assessment	✓			
Informed consent	✓			
Demographics	✓			
Allocation		✓		
Interventions (the intervention will be delivered over a 14‑d period, with treatment administered on 10 d [5 d on, followed by 2 d off])				
taVNS^c^				
Sham taVNS				
Assessments				
PAS^d^		✓	✓	
YPR-SRS^e^		✓	✓	
MASA^f^		✓	✓	
Suprahyoid muscle motor-evoked potentials (TMS-MEP^g^)		✓	✓	
Adverse event records		✓	✓	
FOIS^h^		✓	✓	✓
ECG^i^		✓	✓	

a SPIRIT: Standard Protocol Items: Recommendations for Interventional Trials.

b The intervention period will last 14 days, with treatment administered on 10 weekdays over 2 wk. Day 44 represents the 1-mo follow-up visit after completion of the 2-wk intervention.

c taVNS: transcutaneous auricular vagus nerve stimulation.

d PAS: Penetration-Aspiration Scale.

e YPR-SRS: Yale Pharyngeal Residue Severity Rating Scale.

f MASA: Modified Mann Assessment of Swallowing Ability.

g TMS-MEP: transcranial magnetic stimulation motor-evoked potentials.

h FOIS: Functional Oral Intake Scale.

i ECG: electrocardiogram.

### Data Management

Data will be recorded on standardized paper case report forms (CRFs) and transcribed into electronic databases by independent researchers blinded to group allocation. All participant identifiers will be anonymized prior to statistical analysis. Original CRFs will be archived securely in the hospital medical records department for 3 years after study completion, per institutional ethical requirements. Deidentified datasets can be shared with qualified researchers upon reasonable request to the corresponding author [25,26].

### Statistical Analysis

All statistical analyses will be conducted using SPSS (version 26.0; IBM Corporation) and R (version 4.2; R Foundation for Statistical Computing). Two-sided tests with α of .05 are adopted. All randomized participants will be analyzed following the intention-to-treat principle, and missing values will be reported for all variables. Multiple imputation with 20 datasets will be applied to missing outcome data, with complete-case analysis as a sensitivity check.

Continuous data will be summarized as mean (SD) for normally distributed variables or median (IQR) for skewed distributions, and categorical data will be presented as counts and percentages.

The primary end point, the PAS, is treated as continuous quantitative data rather than ordinal categorical data in this study. Although the PAS consists of discrete integer ratings, its ordered numeric scores represent gradual, incremental changes in swallowing penetration and aspiration severity, supporting the use of continuous linear modeling and a corresponding sample size calculation.

Linear mixed-effects models (LMMs) will be applied to all repeated quantitative outcomes, including PAS, the MASA, hyoid bone displacement, hyoid-thyroid approximation distance, TMS-MEP parameters, and the FOIS. Each LMM includes fixed effects of group allocation, assessment time point, group-by-time interaction, and a random intercept for each participant to account for within-subject correlation of repeated measurements. Baseline observations are incorporated as a single time point in the longitudinal model to adjust for interindividual baseline differences in swallowing function.

The repeatedly measured ordinal scale YPR-SRS will be analyzed using ordinal mixed-effects logistic regression, with fixed and random-effects structures identical to those of the LMMs. All mechanistic TMS-MEP indicators are defined as exploratory outcomes without multiplicity correction. Between-group comparisons for categorical safety data will use a chi-square test or Fisher exact test, as appropriate. Blinding efficacy will be quantified using the BBI [27,28], with values of at least zero indicating successful blinding. Interim analyses will be performed every 2 months and only shared with the Data Monitoring Committee (DMC).

### Interim Analyses

Interim analyses will be performed at 2-month intervals, with preliminary results made available solely to the DMC and the principal investigator, who retains ultimate authority regarding trial continuation [29].

### Harms and Safety Monitoring

Patient safety will be monitored throughout the trial by an independent safety officer who has access to unblinded data [30]. Because taVNS can affect heart rate and autonomic function, and patients with stroke are medically vulnerable, the following safety procedures will be implemented.

#### AE Classification and Grading

AEs will be classified according to the Common Terminology Criteria for Adverse Events (CTCAE) version 5.0, graded from 1 (“mild”) to 5 (“death”). All AEs will be recorded in the CRFs, regardless of whether they are considered related to the intervention.

#### Causality Assessment

The relationship between any AE and the intervention will be assessed by the safety officer and principal investigator, categorized as: unrelated, unlikely, possibly, probably, or definitely related.

#### Monitoring Procedures

Vital signs (heart rate, blood pressure, and oxygen saturation) will be monitored before and after each stimulation session. Monthly meetings will be convened to review each participant’s clinical status and evaluate both treatment efficacy and safety.

#### Stopping Rules

The trial will be stopped if any of the following occur:

- Any serious adverse event (SAE) assessed as possibly, probably, or definitely related to taVNS
- Severe AEs (CTCAE grade ≥3) occurring in more than 20% of participants in either group
- Two or more participants in either group experience a cardiac arrhythmia or significant bradycardia (heart rate <50 beats/min) during or immediately after stimulation

#### Emergency Unblinding

In the event of an SAE in which knowledge of the intervention is necessary for participant management, the independent safety officer will perform emergency unblinding. The reason for unblinding will be documented in the participant’s medical record and reported to the ethics committee.

#### Reporting Timeline

All SAEs will be reported to the Research Ethics Committee of the Guangdong Second Hospital of Traditional Chinese Medicine within 24 hours of occurrence. A summary of all AEs (including type, grade, and relatedness) will be included in the annual progress report to the ethics committee.

### Data Monitoring, Auditing, and Quality Control

#### Data Monitoring

An independent safety officer will monitor participant safety throughout the trial, with access to unblinded data if required for safety assessments. A DMC, comprising members of the Clinical Endpoints Committee and the principal investigator, will review source data and medical events based on prespecified outcomes at regular intervals. Data monitoring activities exclusively cover participant safety surveillance, AE review, interim data review, and early trial termination recommendations, and are entirely independent of routine data recording, CRF archiving, and deidentification procedures detailed in the Data Management subsection, which governs only static data storage rather than real-time safety oversight.

Monthly meetings will be convened to review each participant’s clinical status and evaluate both treatment efficacy and safety. The DMC will have access to interim analysis results, as described in the Interim Analyses subsection, and may recommend early trial termination if safety concerns arise.

#### Auditing

The principal investigator will submit an annual progress report to the corresponding author and the Research Ethics Committee. The progress report will include the recruitment status, summary of AEs, and any protocol deviations. The Research Ethics Committee may conduct independent audits of trial conduct as deemed necessary [31].

Independent audits of trial source documents, medical records, and CRFs may be initiated by the institutional ethics committee at any time throughout the trial period. Upon receiving an audit notice, the principal investigator shall organize all trial-related documents (informed consent forms, therapy logs, AE records, and randomization envelopes) for on-site inspection. All identified protocol deviations will be documented with corrective action plans and tracked until full rectification, with audit summaries submitted to the ethics committee within 14 working days.

#### Quality Control

A quality control team, independent of the intervention and assessment teams, will conduct regular and ad hoc monitoring to verify data accuracy. Data collection will be supervised by an independent research assistant to ensure completeness and consistency.

Protocol deviations must be documented by researchers with a full description of the incident, its cause, corrective actions taken, and the signatures of responsible personnel. Noncompliance with the protocol that cannot be resolved may result in termination of the study.

During the statistical analysis phase, 2 independent research assistants will perform double data entry. They will conduct self-verification to identify any discrepancies in the entered data and promptly rectify any detected errors. These assistants will also be responsible for analyzing, evaluating, and reporting the potential impact of any protocol deviations on the final results and conclusions. All quality control and auditing records will be retained for inspection by the ethics committee or regulatory authorities.

## Results

This study was funded in January 2025. Participant recruitment commenced in March 2025 at the Acupuncture and Rehabilitation Department of the Guangdong Second Hospital of Traditional Chinese Medicine. As of June 2026, 28 of the planned 40 participants have been enrolled and randomized. Data collection began in March 2025 and is projected to be completed in October 2026. The first results are expected to be submitted for publication in winter 2026.

This report describes the study protocol; no outcome data are presented at this stage. This protocol follows Version 5.0 of the trial plan, approved on February 27, 2025, and no outcome data had been reviewed or analyzed at the time of submission. Both ethical approval and official trial registration were completed in full prior to the initiation of participant recruitment. We submitted this protocol ahead of full data collection and analysis to uphold transparency and comply with standard best practices for clinical trial reporting.

## Discussion

Dysphagia is a common complication following a stroke. While its pathogenesis remains incompletely understood and no clearly established therapeutic approach has been identified, taVNS has emerged as a promising intervention for the rehabilitation of PSD.

This randomized controlled trial targets patients with PSD in the recovery phase. The clinical efficacy of taVNS will be assessed using well-validated outcomes, including the PAS, pharyngeal residue volume, hyoid bone displacement, and MEPs of the suprahyoid muscles. Furthermore, the protocol incorporates B-mode ultrasonography to quantitatively evaluate the suprahyoid muscle group. By measuring hyoid bone displacement and its approximation to the thyroid cartilage, this imaging technique provides direct and reliable evidence of the muscular dynamics responsible for laryngeal elevation.

Only the cymba conchae and cavum conchae are anatomically innervated by auricular vagal afferent fibers; the scapha region lacks vagal nerve branches, which is the primary rationale for selecting scapha acupoints as sham stimulation sites. Preliminary evidence suggests that stimulation of the vagus nerve–rich conchal areas may modulate the glossopharyngeal-vagal reflex pathway and boost peripheral vagal afferent signals [5]. Such afferent enhancement could potentially elevate excitability within swallowing-related cortical networks, which is hypothesized to improve swallowing coordination and alleviate PSD symptoms [32]. Anatomically, the Heart (CO15) and Kidney (CO10) acupoints are located in the cavum conchae and cymba conchae (conchal area). According to TCM theory, the functional states of the Heart and Kidney, 2 fundamental zang-fu organs, are closely associated with the manifestation of PSD [33]. The Heart governs spirit and regulates somatic motor function; poststroke heart-spirit insufficiency may disrupt neural control of tongue and pharyngeal movements. Auricular Heart point stimulation is hypothesized to nourish heart qi and restore coordinated tongue-pharyngeal activity, consistent with the TCM doctrine that “the heart opens into the tongue”—sufficient heart qi maintains tongue mobility to facilitate swallowing. The Kidney is the root of innate essence, governing marrow production and cerebral nourishment. Depletion of kidney essence may lead to malnutrition of tongue-pharynx meridians and subsequent swallowing dysfunction. The Kidney also modulates salivary secretion; tonifying kidney qi is theorized to normalize fluid secretion and unblock swallowing-related meridians. Given the integrated theoretical advantages of combining TCM meridian theory and auricular vagal neuroanatomy, we selected bilateral Heart and Kidney auricular sites for electrical stimulation. This study has several potential limitations. First, although conventional stimulation parameters were used to ensure patient safety, the optimal parameter set for taVNS in PSD remains to be experimentally established. While 20-Hz stimulation has been associated with improved limb function after stroke, direct evidence supporting its efficacy for swallowing recovery is lacking. Thus, the alternating frequencies of 4 Hz (for 5 s) and 20 Hz (for 10 s) used in this study—with the intensity adjusted to patient tolerance—may not represent the optimal regimen, and the generalizability of the findings should be interpreted with caution [34]. Second, patients with stroke are susceptible to clinical deterioration, comorbidities, cognitive impairment, or communication difficulties, which may delay the detection of health changes and lead to study withdrawal due to unpredictable events, potentially affecting statistical analysis. Third, the relatively small sample size may limit the precision and external validity of the results.

Upon completion of participant recruitment and full data collection, the primary trial outcome manuscript will be reported in accordance with the CONSORT (Consolidated Standards of Reporting Trials) statement extension for nonpharmacological neuromodulation interventions.

In summary, this study aims to examine the efficacy of taVNS in treating PSD and to investigate its underlying mechanisms. The findings are expected to establish a neurophysiological basis for the clinical application of taVNS, addressing current mechanistic gaps and refining strategies for this noninvasive therapy.

## Supplementary material

10.2196/95182 Checklist 1 SPIRIT checklist.

## Abbreviations

AE: adverse event
BBI: Bang Blinding Index
CONSORT: Consolidated Standards of Reporting Trials
CRF: case report form
CTCAE: Common Terminology Criteria for Adverse Events
FEES: Fiberoptic Endoscopic Evaluation of Swallowing
FOIS: Functional Oral Intake Scale
LMM: linear mixed-effects model
MASA: Modified Mann Assessment of Swallowing Ability
MEP: motor-evoked potential
PAS: Penetration-Aspiration Scale
PSD: poststroke dysphagia
RMT: resting motor threshold
SAE: serious adverse event
SPIRIT: Standard Protocol Items: Recommendations for Interventional Trials
taVNS: transcutaneous auricular vagus nerve stimulation
TCM: traditional Chinese medicine
TMS: transcranial magnetic stimulation
TMS-MEP: transcranial magnetic stimulation motor-evoked potential
YPR-SRS: Yale Pharyngeal Residue Severity Rating Scale
