# Supplementary material for: Efficacy and Mechanism of Transcutaneous Auricular Vagus Nerve Stimulation in Patients With Poststroke Dysphagia: Protocol for a Randomized, Sham-Controlled Clinical Trial
Source: JMIR Res Protoc. 2026 Oct 28;15:e95182. doi: 10.2196/95182 (PMC13715380; doi:10.2196/95182)
Supplement: Supplementary file 1 [file resprot-v15-e95182-s001.docx]

SPIRIT Checklist for our Trials

***Protocol Title:*** ***Efficacy and Mechanism of Transcutaneous Auricular Vagus Nerve Stimulation in Patients with Post-Stroke Dysphagia: protocol for a*** ***randomized, sham-controlled clinical trial***

| **Administrative information** |  |  | **Reporting Item** | **Location in Manuscript** | **Reason if not applicable** |
| --- | --- | --- | --- | --- | --- |
|  | | | | | |
| Title | | [#1](https://www.goodreports.org/reporting-checklists/spirit/info/#1) | Descriptive title identifying the study design, population, interventions, and, if applicable, trial acronym | **Title page** |  |
| Trial registration | | [#2a](https://www.goodreports.org/reporting-checklists/spirit/info/#2a) | Trial identifier and registry name. If not yet registered, name of intended registry | **Abstract** (Trial registration)**; Ethical considerations** |  |
| Trial registration: data  set | | [#2b](https://www.goodreports.org/reporting-checklists/spirit/info/#2b) | All items from the World Health Organization Trial Registration Data Set | Included in registry entry (ChiCTR2500096311) |  |
| Protocol version | | [#3](https://www.goodreports.org/reporting-checklists/spirit/info/#3) | Date and version identifier | **Results** ("Version 5.0, approved on February 27, 2025") |  |
| Funding | | [#4](https://www.goodreports.org/reporting-checklists/spirit/info/#4) | Sources and types of financial, material, and other support | **Funding** |  |
| Roles and responsibilities: contributorship | | [#5a](https://www.goodreports.org/reporting-checklists/spirit/info/#5a) | Names, affiliations, and roles of protocol contributors | **Title page** (authors)**; Authors' contributions** |  |
| Roles and responsibilities: sponsor  contact information | | [#5b](https://www.goodreports.org/reporting-checklists/spirit/info/#5b) | Name and contact information for the trial sponsor | **Ethical considerations** (Guangdong Second Hospital of Traditional Chinese Medicine) |  |
| Roles and responsibilities: sponsor and funder | | [#5c](https://www.goodreports.org/reporting-checklists/spirit/info/#5c) | Role of study sponsor and funders, if any, in study design; collection, management, analysis, and interpretation of data; writing of the report; and the decision to submit the report for publication, including whether they will have ultimate authority over any of these activities. | **Roles and responsibilities: Funder** |  |
| Roles and responsibilities: committees | | [#5d](https://www.goodreports.org/reporting-checklists/spirit/info/#5d) | Composition, roles, and responsibilities of the coordinating centre, steering committee, endpoint adjudication committee, data management team, and other individuals or groups overseeing the trial, if applicable (see Item 21a for data monitoring committee) | **Roles and responsibilities: Clinical Endpoints Committee; Steering Committee; Data monitoring, auditing, and quality control** |  |

| **Introduction** | | | | |
| --- | --- | --- | --- | --- |
| Background and rationale | [#6a](https://www.goodreports.org/reporting-checklists/spirit/info/#6a) | Description of research question and justification for undertaking the trial, including summary of relevant studies (published and unpublished) examining benefits and harms for each intervention | **Introduction** (paragraphs 1–5) |  |
| Background and  rationale: choice of comparators | [#6b](https://www.goodreports.org/reporting-checklists/spirit/info/#6b) | Explanation for choice of comparators | **Introduction** (comparison to Wang et al.; active sham rationale) |  |
| Objectives | [#7](https://www.goodreports.org/reporting-checklists/spirit/info/#7) | Specific objectives or hypotheses | **Objectives** |  |
| Trial design | [#8](https://www.goodreports.org/reporting-checklists/spirit/info/#8) | Description of trial design including type of trial (eg, parallel group, crossover, factorial, single group), allocation ratio, and framework (eg, superiority, equivalence, non-inferiority, exploratory) | **Trial Design** |  |
| **Methods: Participants, interventions, and outcomes** | | | | |
| Study setting | [#9](https://www.goodreports.org/reporting-checklists/spirit/info/#9) | Description of study settings (eg, community clinic, academic hospital) and list of countries where data will be collected. Reference to where list of study sites can be obtained | **Participants: Study Setting** |  |
| Eligibility criteria | [#10](https://www.goodreports.org/reporting-checklists/spirit/info/#10) | Inclusion and exclusion criteria for participants. If applicable, eligibility criteria for study centres and individuals who will perform the interventions (eg, surgeons, psychotherapists) | **Participants: Inclusion Criteria; Exclusion Criteria** |  |
| Interventions: description | [#11a](https://www.goodreports.org/reporting-checklists/spirit/info/#11a) | Interventions for each group with sufficient detail to allow replication, including how and when they will be administered | **Interventions: Standard therapy; taVNS and sham stimulations** |  |
| Interventions: modifications | [#11b](https://www.goodreports.org/reporting-checklists/spirit/info/#11b) | Criteria for discontinuing or modifying allocated interventions for a given trial participant (eg, drug dose change in response to harms, participant request, or improving / worsening disease) | **Participants: Discontinuation criteria** |  |

| Interventions: adherance | [#11c](https://www.goodreports.org/reporting-checklists/spirit/info/#11c) | Strategies to improve adherence to intervention protocols, and any procedures for monitoring adherence (eg, drug tablet return; laboratory tests) | **Interventions: Standard therapy** (adherence ≥ 80%)**; Data monitoring, auditing, and quality control** |  |
| --- | --- | --- | --- | --- |
| Interventions: concomitant care | [#11d](https://www.goodreports.org/reporting-checklists/spirit/info/#11d) | Relevant concomitant care and interventions  that are permitted or prohibited during the trial | **Interventions: Standard therapy** (all components provided identically to both groups) |  |
| Outcomes | [#12](https://www.goodreports.org/reporting-checklists/spirit/info/#12) | Primary, secondary, and other outcomes, including the specific measurement variable (eg, systolic blood pressure), analysis metric (eg, change from baseline, final value, time to event), method of aggregation (eg, median, proportion), and time point for each outcome. Explanation of the clinical relevance of chosen efficacy and harm outcomes is strongly recommended | **Outcomes: Primary outcome; Secondary outcomes; Outcome assessment procedures** (primary time point: day 14) |  |
| Participant timeline | [#13](https://www.goodreports.org/reporting-checklists/spirit/info/#13) | Time schedule of enrolment, interventions (including any run-ins and washouts), assessments, and visits for participants. A schematic diagram is highly recommended (see Figure) | **Table 2** (SPIRIT schedule) |  |
| Sample size | [#14](https://www.goodreports.org/reporting-checklists/spirit/info/#14) | Estimated number of participants needed to achieve study objectives and how it was determined, including clinical and statistical assumptions supporting any sample size calculations | **Sample size calculation** |  |
| Recruitment | [#15](https://www.goodreports.org/reporting-checklists/spirit/info/#15) | Strategies for achieving adequate participant  enrolment to reach target sample size | **Participants: Study Setting** |  |
| **Methods: Assignment of interventions (for controlled trials)** | | | | |
| Allocation: sequence generation | [#16a](https://www.goodreports.org/reporting-checklists/spirit/info/#16a) | Method of generating the allocation sequence (eg, computer-generated random numbers), and list of any factors for stratification. To reduce predictability of a random sequence, details of any planned restriction (eg, blocking) should be provided in a separate document that is unavailable to those who enroll participants or assign interventions | **Randomization** (computer-generated randomization sequence) |  |

| Allocation concealment mechanism | [#16b](https://www.goodreports.org/reporting-checklists/spirit/info/#16b) | Mechanism of implementing the allocation sequence (eg, central telephone; sequentially numbered, opaque, sealed envelopes), describing any steps to conceal the sequence until interventions are assigned | **Randomization** (opaque sealed envelopes, independent researcher) |  |
| --- | --- | --- | --- | --- |
| Allocation: implementation | [#16c](https://www.goodreports.org/reporting-checklists/spirit/info/#16c) | Who will generate the allocation sequence, who will enrol participants, and who will assign participants to interventions | **Randomization** |  |
| Blinding (masking) | [#17a](https://www.goodreports.org/reporting-checklists/spirit/info/#17a) | Who will be blinded after assignment to interventions (eg, trial participants, care providers, outcome assessors, data analysts),  and how | **Blinding** (participants, outcome assessors, statistical analysts blinded; therapists unblinded) |  |
| Blinding (masking):  emergency unblinding | [#17b](https://www.goodreports.org/reporting-checklists/spirit/info/#17b) | If blinded, circumstances under which unblinding is permissible, and procedure for revealing a participant’s allocated intervention during the trial | **Blinding** (emergency unblinding by independent safety officer for SAEs) |  |
| **Methods: Data collection, management, and analysis** | | | | |
| Data collection plan | [#18a](https://www.goodreports.org/reporting-checklists/spirit/info/#18a) | Plans for assessment and collection of outcome, baseline, and other trial data, including any related processes to promote data quality (eg, duplicate measurements, training of assessors) and a description of study instruments (eg, questionnaires, laboratory tests) along with their reliability and validity, if known. Reference to where data collection forms can be found, if not in the protocol | **Outcome assessment procedures; Data collection; Data management** |  |
| Data collection plan: retention | [#18b](https://www.goodreports.org/reporting-checklists/spirit/info/#18b) | Plans to promote participant retention and complete follow-up, including list of any outcome data to be collected for participants who discontinue or deviate from intervention protocols | **Participants: Dropout criteria; Provisions for post-trial care** |  |
| Data management | [#19](https://www.goodreports.org/reporting-checklists/spirit/info/#19) | Plans for data entry, coding, security, and storage, including any related processes to promote data quality (eg, double data entry; range checks for data values). Reference to where details of data management procedures  can be found, if not in the protocol | **Data management** |  |

| Statistics: outcomes | [#20a](https://www.goodreports.org/reporting-checklists/spirit/info/#20a) | Statistical methods for analysing primary and secondary outcomes. Reference to where other details of the statistical analysis plan can  be found, if not in the protocol | **Statistical analysis** (ANCOVA for primary outcome; mixed-effects models for longitudinal outcomes; ordinal logistic regression for ordinal outcomes) |  |
| --- | --- | --- | --- | --- |
| Statistics: additional  analyses | [#20b](https://www.goodreports.org/reporting-checklists/spirit/info/#20b) | Methods for any additional analyses (eg, subgroup and adjusted analyses) | **Statistical analysis; Transcranial Magnetic Stimulation...** (TMS--MEP) (subgroup analyses mentioned) |  |
| Statistics: analysis population and missing data | [#20c](https://www.goodreports.org/reporting-checklists/spirit/info/#20c) | Definition of analysis population relating to protocol non-adherence (eg, as randomised analysis), and any statistical methods to handle missing data (eg, multiple imputation) | **Statistical analysis** (intention-to-treat; multiple imputation with 20 datasets; complete-case sensitivity) |  |
| **Methods: Monitoring** | | | | |
| Data monitoring: formal committee | [#21a](https://www.goodreports.org/reporting-checklists/spirit/info/#21a) | Composition of data monitoring committee (DMC); summary of its role and reporting structure; statement of whether it is independent from the sponsor and competing interests; and reference to where further details about its charter can be found, if not in the protocol. Alternatively, an explanation of why a DMC is not needed | **Data monitoring, auditing, and quality control** (DMC comprising Clinical Endpoints Committee members and PI) |  |
| Data monitoring: interim analysis | [#21b](https://www.goodreports.org/reporting-checklists/spirit/info/#21b) | Description of any interim analyses and stopping guidelines, including who will have access to these interim results and make the final decision to terminate the trial | **Interim analyses** (every 2 months; shared only with DMC and PI) |  |
| Harms | [#22](https://www.goodreports.org/reporting-checklists/spirit/info/#22) | Plans for collecting, assessing, reporting, and managing solicited and spontaneously reported adverse events and other unintended effects of trial interventions or trial conduct | **Harms and safety monitoring** (CTCAE v5.0 grading; causality assessment; stopping rules; SAE reporting within 24 hours) |  |
| Auditing | [#23](https://www.goodreports.org/reporting-checklists/spirit/info/#23) | Frequency and procedures for auditing trial conduct, if any, and whether the process will be independent from investigators and the sponsor | **Data monitoring, auditing, and quality control** (Auditing subsection; annual progress report; ethics committee may conduct independent audits) |  |
| **Ethics and dissemination** | | | | |
| Research ethics approval | [#24](https://www.goodreports.org/reporting-checklists/spirit/info/#24) | Plans for seeking research ethics committee / institutional review board (REC / IRB) approval | **Ethical considerations** (approval obtained: Y202411-003-01) |  |
| Protocol amendments | [#25](https://www.goodreports.org/reporting-checklists/spirit/info/#25) | Plans for communicating important protocol modifications (eg, changes to eligibility criteria, outcomes, analyses) to relevant parties (eg, investigators, REC / IRBs, trial participants, trial registries, journals, regulators) | **Ethical considerations** (unanimous team agreement; registry update) |  |
| Consent or assent | [#26a](https://www.goodreports.org/reporting-checklists/spirit/info/#26a) | Who will obtain informed consent or assent from potential trial participants or authorised surrogates, and how (see Item 32) | **Ethical considerations** (researchers explain; written informed consent obtained) |  |
| Consent or assent: ancillary studies | [#26b](https://www.goodreports.org/reporting-checklists/spirit/info/#26b) | Additional consent provisions for collection and use of participant data and biological specimens in ancillary studies, if applicable | **Ethical considerations** (data confidentiality; de-identified datasets available on request) |  |
| Confidentiality | [#27](https://www.goodreports.org/reporting-checklists/spirit/info/#27) | How personal information about potential and enrolled participants will be collected, shared, and maintained in order to protect confidentiality before, during, and after the trial | **Ethical considerations** (data anonymized; archived for 3 years) |  |
| Declaration of interests | [#28](https://www.goodreports.org/reporting-checklists/spirit/info/#28) | Financial and other competing interests for  principal investigators for the overall trial and each study site | **Conflicting interests** |  |
| Data access | [#29](https://www.goodreports.org/reporting-checklists/spirit/info/#29) | Statement of who will have access to the final trial dataset, and disclosure of contractual agreements that limit such access for investigators | **Ethical considerations** (data archived by PI; available from corresponding author on reasonable request)**; Data Availability** |  |
| Ancillary and post trial care | [#30](https://www.goodreports.org/reporting-checklists/spirit/info/#30) | Provisions, if any, for ancillary and post-trial care, and for compensation to those who suffer harm from trial participation | **Provisions for post-trial care** |  |
| Dissemination policy: trial results | [#31a](https://www.goodreports.org/reporting-checklists/spirit/info/#31a) | Plans for investigators and sponsor to communicate trial results to participants, healthcare professionals, the public, and other relevant groups (eg, via publication, reporting in results databases, or other data sharing arrangements), including any publication restrictions | **Provisions for post-trial care** (detailed report to participants); **Discussion** (CONSORT reporting) |  |
| Dissemination policy: authorship | [#31b](https://www.goodreports.org/reporting-checklists/spirit/info/#31b) | Authorship eligibility guidelines and any intended use of professional writers | **Authors' contributions** (ICMJE criteria implied)**; Acknowledgements** (no AI used) |  |
| Dissemination policy: reproducible research | [#31c](https://www.goodreports.org/reporting-checklists/spirit/info/#31c) | Plans, if any, for granting public access to the full protocol, participant-level dataset, and statistical code | **Data Availability** (available from corresponding author on reasonable request) |  |
| **Appendices** | | | | |
| Informed consent materials | [#32](https://www.goodreports.org/reporting-checklists/spirit/info/#32) | Model consent form and other related documentation given to participants and authorised surrogates | Not included in manuscript; to be provided as separate file | The informed consent form is available in Chinese. |
| Biological specimens | [#33](https://www.goodreports.org/reporting-checklists/spirit/info/#33) | Plans for collection, laboratory evaluation, and storage of biological specimens for genetic or molecular analysis in the current trial and for  future use in ancillary studies, if applicable | Not applicable (no biological specimens collected in this trial) |  |
